# Long-term oxidization and phase transition of InN nanotextures

**DOI:** 10.1186/1556-276X-6-387

**Published:** 2011-05-16

**Authors:** Evangelia Sarantopoulou, Zoe Kollia, Goran Dražic, Spomenka Kobe, Nicolaos Spyropoulos Antonakakis

**Affiliations:** 1 National Hellenic Research Foundation, Theoretical and Physical Chemistry Institute, 48 Vassileos Constantinou Avenue, Athens 11635, Greece; 2 Josef Stefan Institute, Department of Nanostructured Materials, Jamova 39, 1000 Ljubljana, Slovenia

## Abstract

The long-term (6 months) oxidization of hcp-InN (wurtzite, InN-w) nanostructures (crystalline/amorphous) synthesized on Si [100] substrates is analyzed. The densely packed layers of InN-w nanostructures (5-40 nm) are shown to be oxidized by atmospheric oxygen via the formation of an intermediate amorphous In-O_*x*_-N_*y *_(indium oxynitride) phase to a final bi-phase hcp-InN/bcc-In_2_O_3 _nanotexture. High-resolution transmission electron microscopy, energy-dispersive X-ray spectroscopy, electron energy loss spectroscopy and selected area electron diffraction are used to identify amorphous In-O_*x*_-N_*y *_oxynitride phase. When the oxidized area exceeds the critical size of 5 nm, the amorphous In-O_*x*_-N_*y *_phase eventually undergoes phase transition via a slow chemical reaction of atomic oxygen with the indium atoms, forming a single bcc In_2_O_3 _phase.

## Introduction

Recent investigations reveal that oxygen contamination plays the most prominent, though not the only role for optimum semiconducting and optical properties of InN films [1-3]. Oxidization is a constraint of using InN in a variety of applications, such as solar cells, charge memories, and light emitting diodes, despite its excellent semiconducting properties. Furthermore, the long-term functionality of InN films is directly related to the growing methodologies because the oxidization rate depends on the morphology and the quality of the InN surfaces [4]. However, reactive sputtering of InN films in oxygen indicated no evidence of direct formation of In_2_O_3_. It was suggested therefore that oxygen accelerates the formation of an intermediate amorphous indium oxynitride phase inside the InN matrix, which eventually oxidizes InN completely to form In_2_O_3 _[5]. This argument was further supported by the fact that immediate oxidization of InN films was observed at temperatures higher than 600 K but not at room temperature [6].

The oxidization process of InN films in air at room temperatures was investigated previously using X-ray diffraction, X-ray photoelectron spectroscopy [7], and high-resolution transmission electron microscopy (HRTEM) [8,9].

However, few data are currently available in the literature for the long-term oxidization (aging) process of InN films at ambient conditions [10]. Recent investigations of long-term oxidization of hcp-InN lattices grown on GaN/sapphire substrates indicate a phase transformation of hcp-InN to an alloy phase of fcc-InN (InN-zb) and fcc-In_2_O_3_, (InN-zb/In_2_O_3_), after exposing the films for 36 months at ambient conditions [8]. The above studies suggested the formation of an intermediate amorphous In-O_*x*_-N_*y *_phase, but a direct observation of this phase has not been reported yet in the literature, nor the phase transformation process was analyzed in relation to the dimensions of the nanodomains.

Among the various growth techniques of nitride films, pulsed laser deposition (PLD) has been employed to grow high-quality nitride films, with the advantage of eliminating either the need for substrate's nitridation or for the formation of an intermediate amorphous buffer layer. This approach was feasible because PLD was carried out in a less-reactive atmosphere in comparison to different methodologies [11]. PLD with a KrF laser, both in vacuum and in nitrogen, was used for depositing InN film on a sapphire substrate [12]. Furthermore, the InN crystalline quality and the electron mobility of InN films grown on c-plane sapphire substrates in nitrogen at 248 nm were highly correlated with the substrate's surface nitridation [13,14].

Growing the nitride films by PLD at 157 nm (molecular fluorine laser) is advantageous because it ionizes the target material and the ambient gases, such as nitrogen, with only one or two photons. Owing to the high energy per photon at 157 nm (7.9 eV), relatively lower laser energy and flux are required to ablate the target in comparison with longer laser wavelengths [15-17]. The nitride molecular structures are therefore formed at lower plasma temperature, either in the substrate or in the plume. Molecular species with small dissociation energy such as the InN molecules are efficiently formed at cooler laser plasma. Good quality bi-phase nitride nanospheroid and crystal structures grown by PLD at 157 nm were demonstrated recently [18,19].

In this article, the long-term oxidization of InN from the atmospheric oxygen at the nanoscale level and the correlation between the oxidization rate and the size of the InN nanostructures are analyzed. InN-w nanocrystals (5-40 nm) surrounded by amorphous nanodomains are grown on Si [100] substrates from an indium target by 157 nm PLD in nitrogen. HRTEM, energy-dispersive X-ray spectroscopy (EDXS), electron energy loss spectroscopy (EELS), and selected area electron diffraction (SAED) analysis are employed to analyze the oxidization process.

This article is organized as follows: In "Experimental procedure" section, the experimental conditions and the analytic methodologies are presented. The morphology of the InN nanostructures is presented in sections "Morphology of InN films" and "High resolution images of InN nanostructures." The structure and the morphology of long-term-oxidized InN nanostructures are given in section "Structure and morphology of long-term-oxidized InN nanostructures" while in section "Amorphous In-O_*x*_-N_*y *_and In_2_O_3 _nanostructures," the relations among the amorphous In-O_*x*_-N_*y*_, the crystalline InN, and In_2_O_3 _phases are discussed and analyzed.

## Experimental procedure

**Figure 1:**
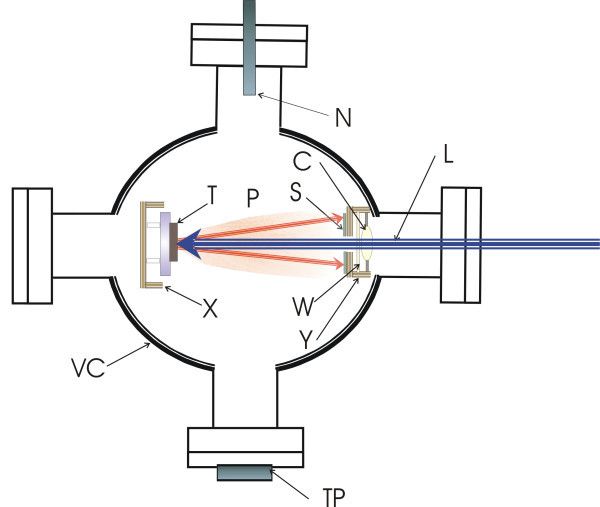
**Schematic diagram of the PLD configuration**. *VC*, stainless steel vacuum chamber; *L*, 157-nm laser beam; *X*, *x*-*y*-*z*-computer-controlled translation stage; *T*, high-purity indium foil; *P*, ablation plume; *C*, CaF_2 _focusing optics; *W*, CaF_2 _window; *S*, Si [100] substrate; *Y*, substrate holder stage; *N*, nitrogen inlet; *TP*, turbo molecular pump.

The apparatus for the PLD experiment consists of a molecular fluorine laser at 157 nm [20-23] (Lambda-Physik, LPF 200), a stainless steel vacuum chamber, a computer-controlled *x*-*y*-*z *translation stage, the focusing optics, and a holder with the Si [100] substrate onto which the indium nitride films are deposited (Figure 1).

The ablative targets are of high-purity (99.999%) indium foils (Good-Fellow) of 1.0-mm thickness. These are mounted inside the chamber on a motorized *x*-*y*-*z *stage, allowing alignment with the beam by external control. The target is oriented normal to the axis of the incident beam.

Films are deposited on Si [100] substrates at the relatively low laser energy of 20 mJ at 15-Hz repetition rate. The pulse duration is 15 ns at full width at half maximum. The laser beam is focused on the target along the laser beam direction with a CaF_2 _lens of 5-cm focal length to a 200-μm spot. The intensity of the focused beam on the target is approximately 6 TW. The Si [100] substrate is placed approximately 3-5 mm away from the target and perpendicular to the optical axis of the laser beam (Figure 1). Before deposition, the vacuum chamber was evacuated down to 10^-4 ^Pa under heating to remove contaminants on the chamber's inner surfaces. The film is deposited at 10^5 ^Pa in nitrogen gas at ambient temperature. Research grade N_2 _(99.999%) is flooded into the chamber before irradiation. The CaF_2 _lens is protected from the ablative products by a 1-mm-thin CaF_2 _window that needs to be replaced after 10 h. The laser beam is focused along the *Z*-direction with micro resolution. The growth rate of the film is 170 nm/h. By changing the laser energy, the background gas pressure, the target-substrate distance and the repetition rate of the laser, films of different morphologies are grown [24,25].

Scanning electron microscopy (SEM, Jeol 840A), EDXS and atomic force microscopy (AFM, Quesant Nomad) are employed to evaluate the morphology and roughness of the films. The structure of the film is studied using a Jeol 2010 FEG scanning transmission electron microscope and a Jeol 2100 transmission electron microscope (TEM) operating at 200 kV, with EELS compositional analysis capabilities.

Small fragments of InN films are transferred to a lacey-carbon-coated Ni grid and examined in the microscope. HRTEM and SAED have been employed to examine the nanostructure and crystalline quality of the InN films. HRTEM measurements are carried out to study the lattice image structural relationship between the hexagonal InN and cubic In_2_O_3 _nanostructures.

## Results and discussion

### Morphology of InN films

**Figure 2:**
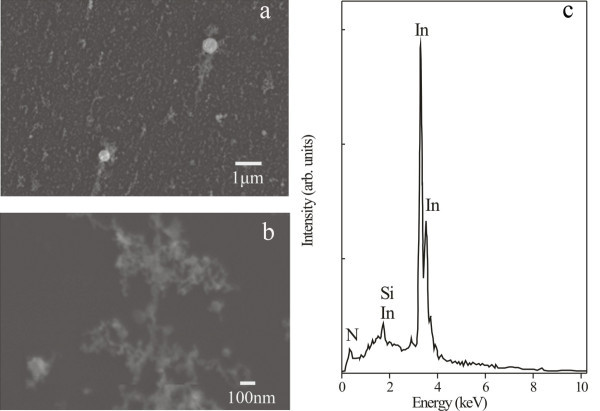
**SEM image of InN film**. **(a) **SEM image of an InN film deposited on a Si substrate showing the aggregation of dendrite-like structures. Two spherical droplets are indicated on the film surface. **(b) **SEM image of the InN film deposited on a Si substrate showing the dendrite fractal structure. **(c) **EDXS of a droplet indicating that InN is formed in the plume.

A microscale SEM image of an InN film deposited on a Si substrate with 1.2 × 10^4 ^laser pulses in nitrogen (10^5 ^Pa) is shown in Figure 2a. The InN composites aggregate into dendrite nanostructures (Figure 2b). Besides the fractal structure, some spherical droplets of InN or indium are scattered on the substrate (Figure 2a). The indium droplets are ejected from the indium target because of its relatively low melting point. The efficiency of droplet's formation depends on the experimental conditions. For example, the formation of InN droplets by metalorganic vapor phase epitaxy or metalorganic chemical vapor deposition was due to indium surface segregation when the ammonia concentration was insufficient [26,27]. Once a droplet attains its critical size, it becomes thermodynamically stable and continues to grow [18]. At the same time, efficient nitridation of droplets is taking place either on the target's surface or in the plume following laser ablation at 157 nm [17-19].

The InN structure was first identified by EDXS, (Figure 2c), recorded 2 days after the growth of film. The spectrum indicates the presence of either the amorphous or crystalline InN [28]. Oxygen was non-detectable, confirming the low oxidization rate at 2 days after the growth of film.

The growth of InN crystals by 157-nm PLD sidesteps some constraints imposed by the low reactivity of nitrogen. The InN crystalline phase is formed either through the ionic interaction of the molecular species on the target, or in the plume near the target's surface. Indeed, two 157-nm photons are required to ionize the N_2 _molecule from its ground electronic state. This condition is satisfied directly on the target because of the high-intensity-focused laser beam.

**Figure 3:**
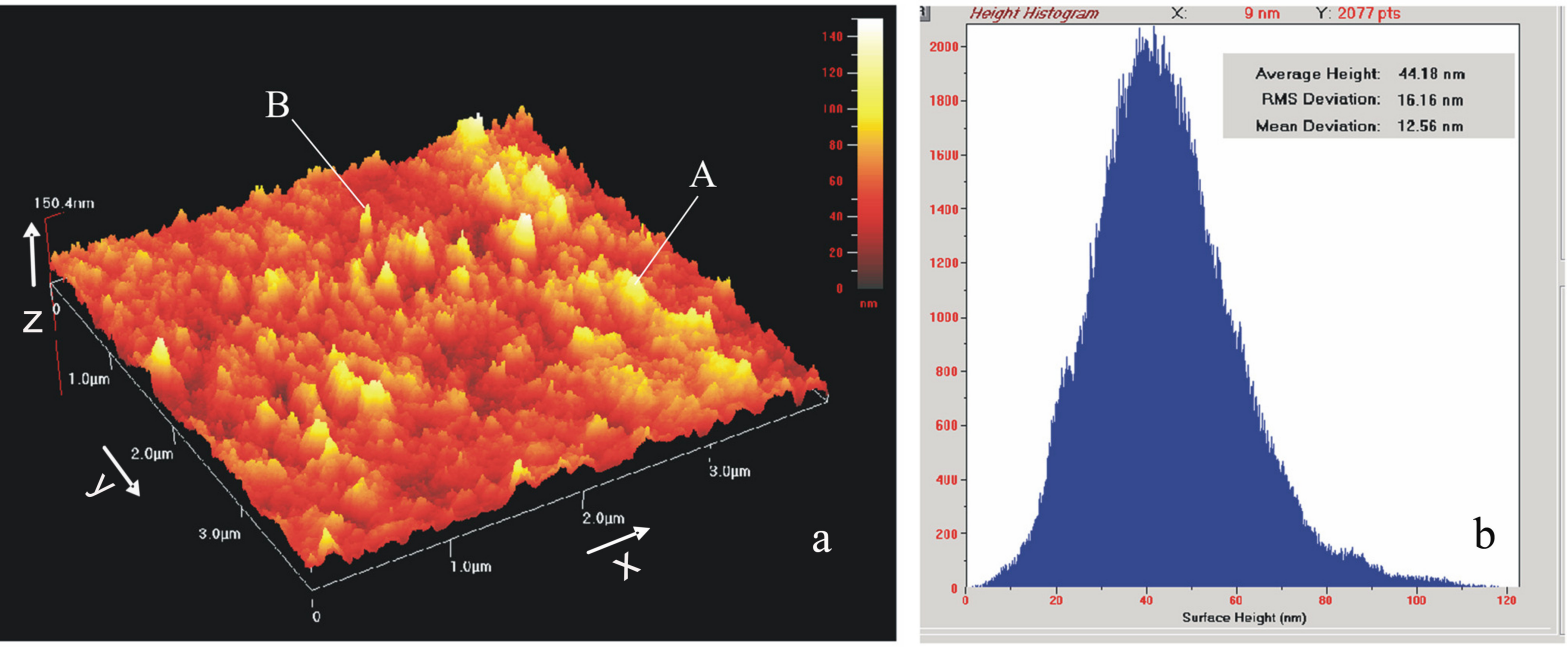
**AFM surface image of InN films deposited on Si substrate**. **(a) **AFM image of the InN film. **(b) **Size distribution histogram of the InN film.

At longer film deposition times (2 h, approx. 10^5 ^laser pulses, 20 mJ per pulse), the morphology of the InN film appears to be denser and thicker, with two distinct morphological features (Figure 3a). The first feature type represents irregular dendrites (Figure 3a, A), while the second feature represents nanorod-like structures (Figure 3a, B), with mean width and height being 40 and 50 nm, respectively, Figure 3b. The nanorods are growing on the top of 100-nm long (*Z*-direction) three-dimensional dendrite islands. The surface roughness distribution (*Z*-direction) (Figure 3b) is asymmetric for films grown for longer period of time. The surface roughness distribution has a major peak at 44 nm and a secondary one at 85 nm.

Irregular-shaped grains observed previously [7,29], were associated with nucleated InN hexagonal crystal islands (grown along the *Z*-direction) that eventually were enlarged laterally along the InN  plane. Moreover, in previous studies, it was confirmed that the surface morphology of InN samples was influenced by the lattice mismatch (InN: hexagonal, *a *= 0.354 nm, *c *= 0.570 nm. Si: diamond cubic *a *= 0.543 nm), between the samples and the Si (111) substrate leading to the growth of three-dimensional islands with size and density depending on the substrate temperature [29].

The lattice mismatch is the source of shear stresses at the interface that tailors the morphology of the film [30], e.g., dendrite structure in our case. The InN crystal has a lattice mismatch with most of monocrystalline substrates, and therefore different morphologies are expected to grow on different substrates [31].

### High-resolution images of InN nanostructures

**Figure 4:**
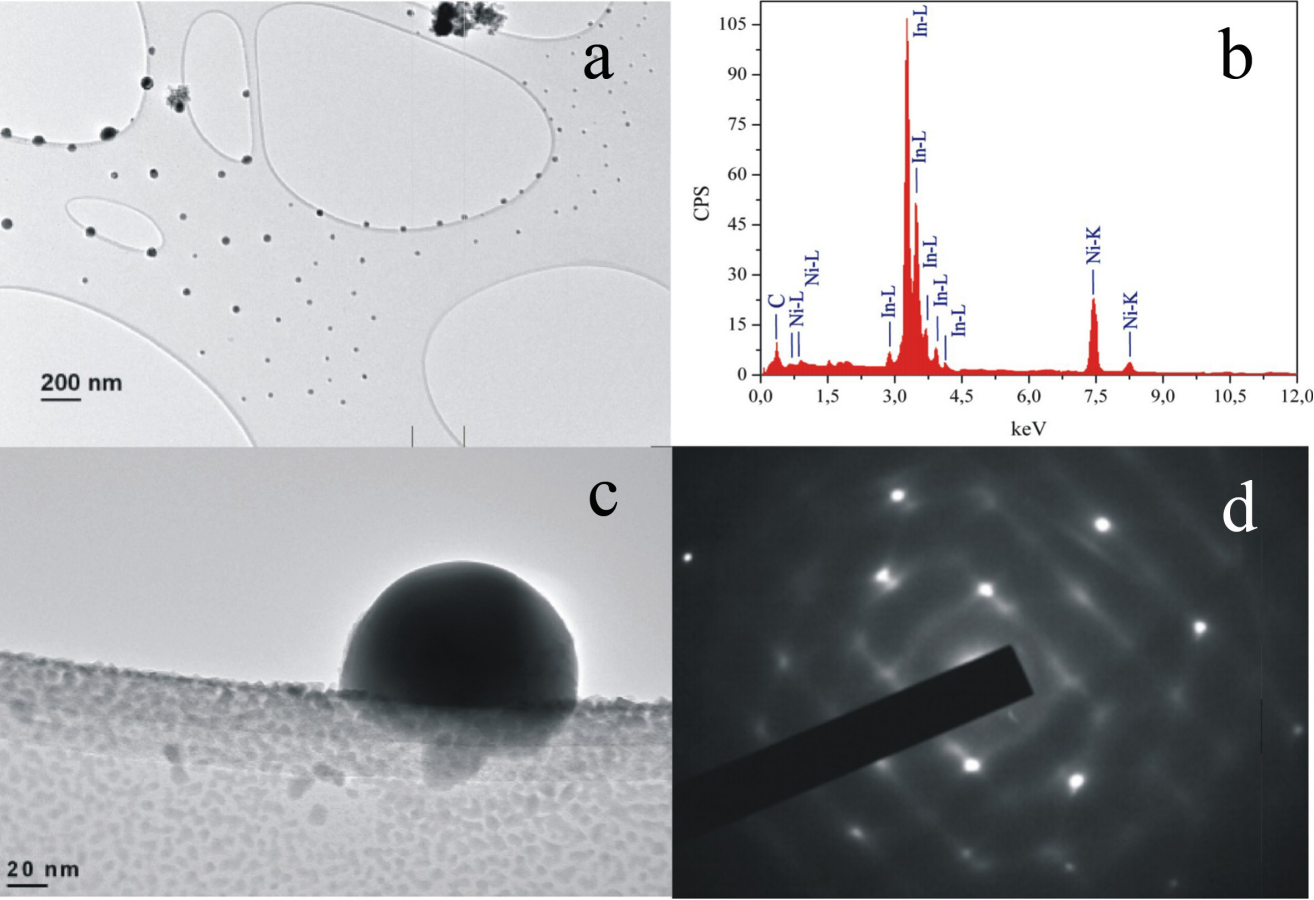
**TEM image and EDXS of InN surface following electron beam impact**. **(a) **TEM image of the destroyed nanocrystalline domains deposited on Ni grids after electron irradiation. **(b) **EDXS of the e-beam-irradiated crystal nanodomains. Only the indium peak is observed. **(c) **Indium nanocrystal sphere of tetragonal structure formed after e-beam irradiation. **(d) **SAED of the nanosphere with the tetragonal crystalline structure of pure indium.

The capturing of HRTEM images of InN crystals is not a trivial issue because InN is unstable under electron irradiation [32], due to its low dissociation energy (0.073 eV) [33]. The InN crystals first are transferred to a lacey-carbon-coated Ni grid using a scalpel knife, and then they are transferred to the carbon-coated TEM grid. The samples are examined under mild electron beam conditions; current density of the order of 10^-5 ^nA/nm^2^, 50-μm condenser aperture, and spot size 3. With this method, although the information of relative orientation between the InN and Si substrate is lost, there are no artifacts such as structure alteration related to ion-milling. In addition, ion-milling is not recommended because InN is thermally dissociated. The electron beam destroys the InN structure by changing the morphology of the dendrite structures, and new aggregations of 20-40 nm nanospheres distributed selectively around the periphery of the grid cells are formed (Figure 4a). The change of the film morphology is accompanied by structural changes and the EDXS of the areas exposed to e-beam and a deficiency in nitrogen (Figure 4b). This is additionally confirmed by SAED, and the image of one indium nanosphere (Figure 4c) reveals the tetragonal crystal structure of pure indium (Figure 4d). In addition, no traces of oxygen are identified.

**Figure 5:**
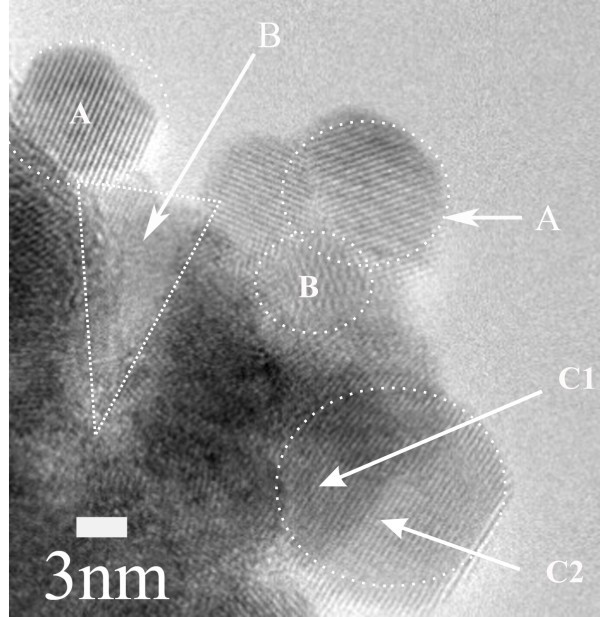
**HRTEM image of InN nanostructures deposited on a Si [100] substrate**. The images indicate the superposition of crystal areas of different contrasts and with parallel Laue levels, depicted within the peripheries of the circles C1, C2 and A. Various amorphous nanodomains are indicated within the peripheries of the triangle and the circle B.

Using mild electron beam conditions two days after the PLD experiment, hexagonal hcp-InN crystal (Figure 5, circle A) and amorphous phases (Figure 5, circle B) approximately 10 nm wide are identified.

The long-term phase transformation from the hcp-InN phase to the metastable fcc-InN structure reported previously [8] is undetectable because of the growth of the film on a different substrate where the lattice mismatch provided the required strain for phase transformation.

### Structure and morphology of long-term-oxidized InN nanostructures

Three different methods are used for specifying the stoichiometric composition of the crystal nanodomains: EDXS, EELS, and SAED because results among these three methods may vary. This inconsistency is not only due to the presence of stray X-ray radiation at EDXS, especially at rough films with broad size distribution of nanodomains but also due to the difference between the cross sections of the electron and the X-ray beams (5 and 450 nm, respectively).

**Figure 6:**
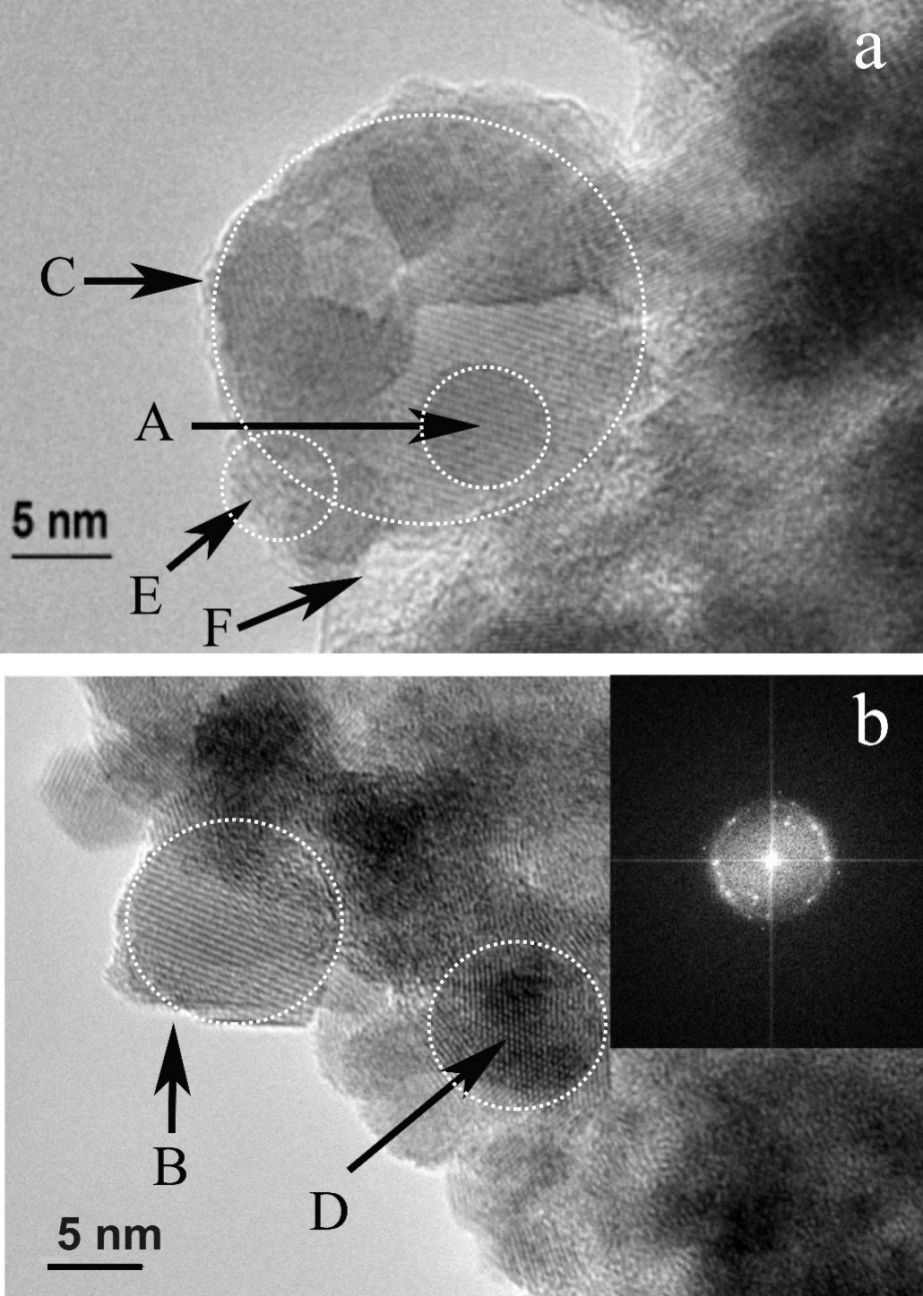
**Morphology of InN nanostructures grown on Si [100] substrate, indicating coexistence of the crystal line InN, In_2_O_3 _and the amorphous oxynitride In-O_*y*_-N_*x *_phases**. **(a) **5-nm crystal domains (A), 40-nm crystal domains (C), amorphous oxynitride phase (E), boundary between amorphous and crystal phase (F). **(b) **Crystal cubic In_2_O_3 _nanodomains. In the inset, the FFT images are indicated. The crystal In_2_O_3 _nanostructure within the circle (B) is 25 nm wide. The In_2_O_3 _crystal nanostructures are rotated with respect to each other (D).

After storing the films for 6 months at ambient conditions, the co-existence of crystal In_2_O_3 _and amorphous In-O_*x*_-N_*y*_, together with the InN crystal phase is identified. The HRTEM images indicate the superposition of crystal areas of different contrast and parallel Laue levels, shown within the periphery of the circles C1, C2, and A in Figure 5 and 5A and 5C in Figure 6a, respectively. The image of Figure 5 is recorded immediately after the growth of the film, while that of Figure 6 after 6 months. It is likely that these images correspond to the growth of 2D crystal structures on the top of each other forming thus 3D structures. The picture resembles the collection of randomly overlaid coins with aligned heads.

This point is further strengthened by previous investigations on the size of monocrystals. It was found that they exhibit a sharp Gaussian distribution at the nanoscale (approx. 10 nm) [34]. It is unlikely therefore that long 3D crystal structures are grown either in the plume, or directly on the Si substrate with PLD. The relatively small size of the crystal nanostructures is credited to the short range of ionic forces that are responsible for the short spatial correlation between the atoms of the crystal structure. Three different nanostructured groups with average size of 5 (A), 25 (B), and 40 nm (C) are identified, Figure 6a, b, although larger crystal domains are grown through the aggregation of small nanocrystal domains (<5 nm). Furthermore, crystal structures with their Laue levels slightly rotated with respect to each other (Figure 6b, D) should correspond to a superposition of uncorrelated crystal nanostructures [34] (separated by a relatively large distance in comparison to the correlation length). The HRTEM images indicate the superposition of crystal areas of different contrast and with parallel Laue levels, indicated within the periphery of the circles C1 and C2 (Figure 5) and within the areas E and F (Figure 6a). Furthermore, various amorphous nanodomains are formed, such as the ones within the periphery of the triangle and the circle B (Figure 5), and the areas within the periphery of the circles E and F (Figure 6a).

The nanoclusters tend to nucleate primarily on the substrate, but the size distribution of nanodomains is an indication that crystallization in the plume is possible too.

**Figure 7:**
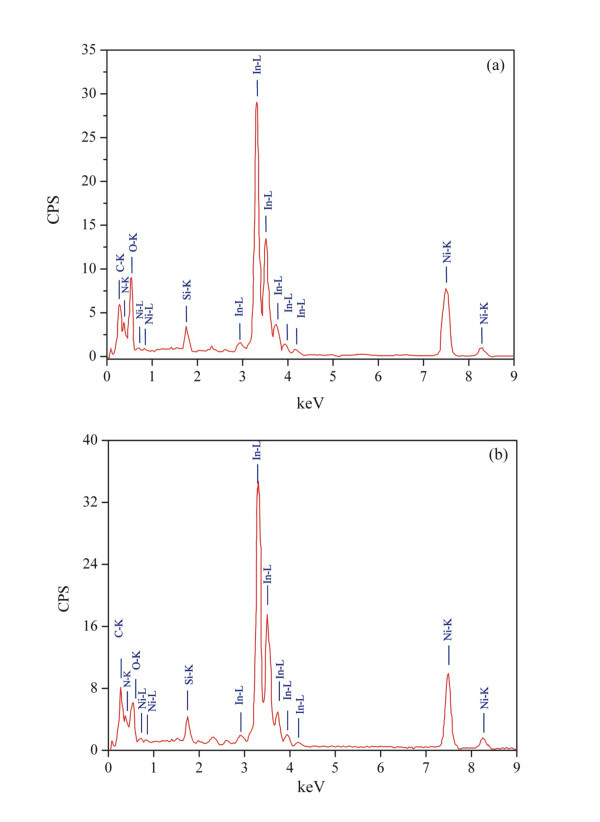
**EDXS of film deposited on a Si substrate**. The ratio of concentrations is (0.5InN/0.5In_2_O_3_) **(a)**, and (0.3InN/0.7In_2_O_3_) **(b) **for 3 and 6 months exposure at ambient conditions, respectively.

The concentration of oxygen in the film, measured with EDXS, is constantly increasing during the storage period and is uniform in areas of the same film morphology while the presence of oxygen is noticeable in the entire film area indicating the post-ablation oxidization process. A typical EDXS recorded 3 and 6 months after the film growth is shown in Figure 7a, b.

The average experimental concentrations of nitrogen and oxygen are affected negligibly as observed from the X-ray absorbance, and the molar ratio of InN/In_2_O_3 _is estimated as 0.3/0.7. Indeed, owing to the MIV and MV absorption edges of In, the mass absorption coefficients (MACs) for O_Kα _and N_Kα _are 1.9 × 10^4 ^and 3.7 × 10^3 ^cm^2^/g, respectively. In addition, owing to the K absorption edge of nitrogen, the MACs of O_Kα _and N_Kα _lines in nitrogen are 1.7 × 10^4 ^and 1.5 × 10^3 ^cm^2^/g, respectively. The calculated attenuation of O_Kα _line in a 10-nm-thick crystal (0.5InN/0.5In_2_O_3_) is approx. 10%, and the attenuation of the N_Kα _line is approx. 2%.

**Figure 8:**
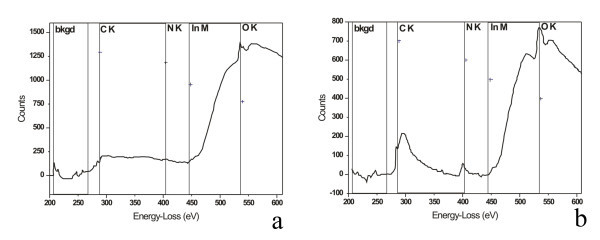
**EELS spectra of nanostructures**. **(a) **EELS spectra of large nanostructures where only oxygen is present. **(b) **EELS spectra of small nanostructures, where both oxygen and nitrogen are present.

In addition, the elemental analysis with EDXS depends on the film morphology, and therefore EELS elemental analysis is used on small (<5 nm), and larger crystal and amorphous domains. The average concentration of elements (mol%) of the small-sized domains with EELS is 40% In, 40% O, and 20% N, respectively (Figure 8a, b). No traces of nitrogen are detected on large-sized domains as can be seen in Figure 6b. These results imply that oxidization starts by forming a small nucleus of the oxidized areas in the InN nanodomains with dimensions less than 5 nm. Taking into consideration that the surface of InN films is highly porous, the atmospheric oxygen diffuses first into the InN grid and then slowly oxidizes InN to an amorphous In-O_*x*_-N_*y *_phase (Figure 6a, C). The amorphous phase eventually undergoes a second-order phase transition to a crystal line In_2_O_3 _phase, when the size of the oxidized areas exceeds the critical value of approx. 5 nm. The results of EELS and HRTEM suggest that the chemical reaction is accompanied by agglomeration of small-sized oxidized domains, forming larger crystal domains of indium oxide only.

**Figure 9:**
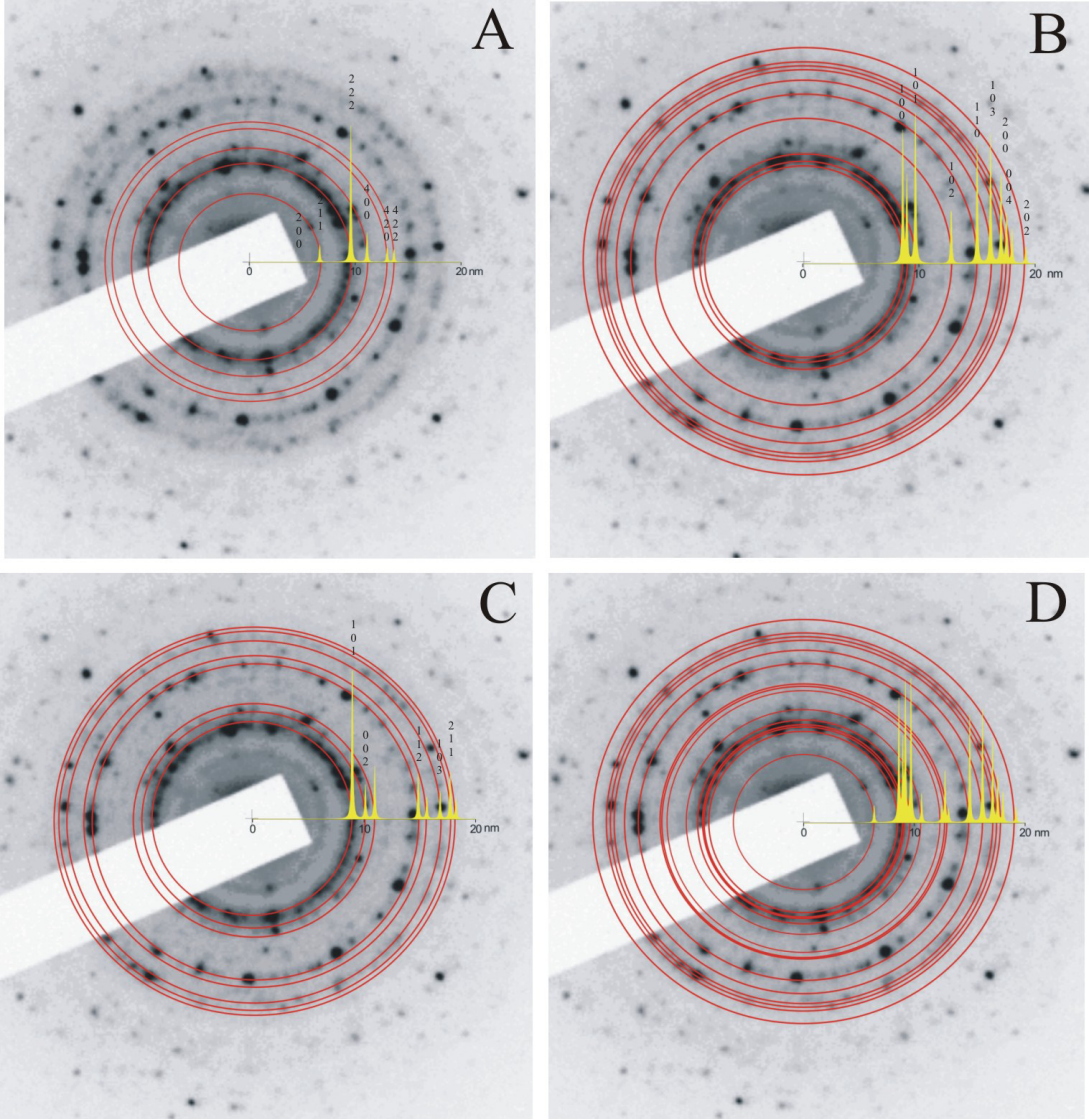
**Calculated intensity-line profile and *d*-spacing (homocentric circle) overlaid on the experimental SAED patterns**. **(a) **Cubic bcc In_2_O_3 _structure, **(b) **hexagonal InN structure, **(c) **tetragonal indium structure, and **(d) **mixture of 50% InN-50% In_2_O_3_.

Besides EDXS and EELS, the levels of oxidization of InN films are measured by SAED for different areas of the samples (Figure 9). The diffraction spots are fitted to the hcp-InN and bcc-In_2_O_3 _phases (the patterns are taken with 450-nm diameter of the electron beam). Taking into consideration the rotational average of the experimental SAED patterns, the intensity distribution of the diffracted electrons is deconvoluted using Diff Tools scripts in Gatan's Digital Micrograph software. Next, SAED intensity distributions for pure hcp-InN and bcc-In_2_O_3 _are calculated using the EMS program code. The best fit between the experimental diffraction patterns and the calculated intensity distributions is obtained by the linear combination approach (sum of weighted parts of both the simulated distributions for InN and In_2_O_3_). After an examination of the samples stored for 6 months in ambient conditions, we found approximately equal concentrations of hcp-InN and bcc-In_2_O_3 _phases (50% InN(hcp)-50% In_2_O_3_(bcc)). The best fit to the experimental patterns is achieved for this concentration, which is in agreement with the EDXS results, where the concentration of oxygen is constantly increasing. When the same sample is stored in isopropanol for the same duration, the best fit is for 80%hcp-InN/20%bcc-In_2_O_3_, confirming once more that oxidization takes place after the growth of film from the atmospheric oxygen.

The SAED images were obtained with fast Fourier transmission (FFT) on HRTEM images. This technique eliminates part of the information from the initial image, while preserving the periodical information, such as the lattice plane fringes. In the experimental SAED pattern, six clear rings made of discrete spots can be fitted (Figure 9).

The experimental SAED patterns are compared to the simulated patterns of the intensity distribution of the diffracted beam (yellow inlets, Figure 9) for three different phases (a, bcc-In_2_O_3_; b, hcp-InN; and c, bcc-In). The experimental SAED patterns are usually slightly distorted because of non-optimized alignment of the microscope and the nonlinearity of the CCD camera.

First, the *d *spacing values are calculated for cubic bcc In_2_O_3 _nanostructures. The poor matching of peak positions between the calculated and the experimental profiles of Figure 9a (bcc-In_2_O_3_) indicates that additional crystalline phases besides bcc-In_2_O_3 _and hcp-InN are present, forming an alloy texture of hcp-InN/bcc-In_2_O_3_, and that a certain amount of oxygen is present in the form of amorphous In_2_O_3 _or In-O_*x*_-N_*y *_non-detectable with SAED. This result is in agreement with the direct identification of the amorphous phase from the HRTEM images (Figure 6E).

The same procedure is applied for pure hexagonal hcp-InN and tetragonal indium. In all the cases, the agreement with the experimental SAED patterns is lacking.

The sum of simulated patterns for 50% hcp-InN and 50% bcc-In_2_O_3 _is in agreement with the experimental ones, indicating the coexistence of cubic bcc-In_2_O_3 _and hexagonal hcp-InN phases in the films (Figure 9d).

### Amorphous In-O_*x*_-N_*y *_and In_2_O_3 _nanostructures

It is generally accepted that InN is transformed first to the amorphous In-O_*x*_-N_*y*_/In_2_O_3 _phase and then subsequently to the crystalline In_2_O_3 _phase either by aging or annealing. It has been reported that after the initial growth of InN and subsequent formation of oxynitride, a mixed crystalline phase of In_2_O_3 _and InN crystals was grown after a short annealing time (10 min) at 550°C [35]. The cubic In_2_O_3 _phase was formed layer-by-layer, after subsequent oxidization of the hexagonal InN layers [36].

Substitution of nitrogen by oxygen is possible in principle, since both atoms have similar atomic radii [37]. At room temperature and up to 800 K, the heat of formation of crystalline In_2_O_3 _(221 kcal/mol) is higher than the heat of formation of crystalline InN (34 kcal/mol) [37], and therefore, even in the presence of oxygen, InN is preferably formed.

In the present study, InN is growing in pure nitrogen, and thus, the formation of In_2_O_3 _during the ablative phase is negligible. Furthermore, the oxidization of the post-ablated pure indium nanodomains from the atmospheric oxygen or the direct transformation of the crystal phase of InN to In_2_O_3 _are improbable to occur because oxidization via those pathways is energetically unfavorable. Following the film's exposure to atmospheric oxygen, the oxidization of InN is taking place slowly. The initially physisorbed oxygen eventually becomes chemisorbed inside the InN lattice. Therefore, the oxidization of the InN crystal should take place through the formation of an intermediate amorphous phase.

The single crystal layers are clearly identified, and the inset of FFT of real images and the experimental results indicate that the crystal In_2_O_3_, InN, and the amorphous In-O_*x*_-N_*y *_oxynitride phases coexist in the form of neighboring distinct nanodomains after the long-term exposure of InN to atmospheric conditions (Figure 6a, F).

## Conclusions

InN-w crystal nanostructures are grown by PLD at 157 nm from an indium target in nitrogen without post-growth annealing or target heating. After exposing the films to oxygen at ambient conditions for 6 months, HRTEM imaging, EDXS, EELS, and SAED analyses reveal that long-term oxidization is taking place slowly through the formation of an intermediate amorphous In-O_*x*_-N_*y *_oxynitride phase, directly identified by HRTEM. The atmospheric oxygen, diffused inside the InN grid, oxidizes the film slowly. This process consists of two stages: first, the intermediate amorphous phase is formed and, when it exceeds the critical size of 5 nm, the In_2_O_3 _crystalline phase is formed via phase transition. Finally, oxidization at a larger scale occurs from the agglomeration of the small-sized-oxidized crystal nanodomains.

## Abbreviations

AFM: atomic force microscopy; EDXS: energy-dispersive X-ray spectroscopy; EELS: electron energy loss spectroscopy; FFT: fast Fourier transmission; HRTEM: high-resolution transmission electron microscopy; MACs: mass absorption coefficients; PLD: pulsed laser deposition; SAED: selected area electron diffraction; SEM: scanning electron microscopy; TEM: transmission electron microscope.

## Competing interests

The authors declare that they have no competing interests.

## Authors' contributions

ES conceived of the study, participated in its design and coordination, drafted the manuscript and carried out the film growth and AFM imaging. ZK participated in the samples preparation and in the AFM morphological characterization and interpretation. GD carried out SEM and HRTEM characterization, EELS and SAED analysis and interpretation of results. SK participated in the designing of experiment, in the discussions and analysis of experimental data. NSA participated in the samples preparation. All authors read and approved the final manuscript.
